# Prospective laparoscopic assessment of sigmoid elongation: association with constipation and abdominal pain

**DOI:** 10.1007/s00423-026-04015-4

**Published:** 2026-03-25

**Authors:** Jamal Driouch, Mustafa Abbas Abdulqader, Shazadi Sajid, Nikolaos Kapellas, Qais Riyal, Metin Senkal

**Affiliations:** 1 Department of Surgery, Elisabeth Hospital, Lehrkrankenhaus of Ruhr University of Bochum, Hochstraße 63, Iserlohn, 58638 Germany; 2 Department of Surgery, Marien Hospital Witten, Lehrkrankenhaus of Ruhr University of Bochum, Marienplatz 2, Witten, 58452 Germany; 3 Department of Surgery, Marien Hospital Herne, Ruhr University of Bochum, Hölkeskampring 40, Herne, 44625 Germany

**Keywords:** Dolichosigma, Constipation, Abdominal pain, Laparoscopy, Sigmoid colon, Colonic redundancy

## Abstract

**Background:**

Constipation is a common gastrointestinal complaint affecting up to 14% of the adult population, with a higher prevalence in women and older individuals. Structural abnormalities such as dolichosigma—elongation of the sigmoid colon—have long been discussed as potential contributors, but prospective intraoperative data in unselected adult populations remain scarce. The absence of standardized anatomical reference values further complicates clinical interpretation.

**Methods:**

In this prospective observational study, sigmoid length was measured intraoperatively under standardized laparoscopic conditions in consecutive adult patients undergoing minimally invasive abdominal surgery. Clinical data including demographic parameters, comorbidities, Wexner Constipation Score (WCS), self-reported constipation, and abdominal pain were recorded. Associations between sigmoid length and symptoms were analyzed, and a multivariable logistic regression model was performed to adjust for potential confounders (age, sex, BMI).

**Results:**

A total of 178 patients were included. Mean sigmoid length was 30.5 ± 12.2 cm (range 10–120 cm). Constipation was present in 37.6% and was associated with longer sigmoid length (32.6 ± 12.9 cm vs. 29.2 ± 11.6 cm; *p* = 0.006) and higher WCS (11.5 ± 5.5 vs. 3.5 ± 3.3; *p* < 0.001). A statistically significant but modest correlation between sigmoid length and WCS was observed (*r* = 0.26; *p* < 0.001). Patients reporting abdominal pain also exhibited longer sigmoid segments (31.4 ± 12.5 cm vs. 27.4 ± 10.9 cm; *p* = 0.031). In multivariable analysis, sigmoid length remained independently associated with constipation (adjusted OR 1.03 per cm, 95% CI 1.01–1.06; *p* = 0.019).

**Conclusion:**

Sigmoid elongation is associated with increased prevalence of constipation symptoms and abdominal pain in an unselected surgical population. A sigmoid length of approximately ≥ 33–35 cm may represent a pragmatic anatomical reference associated with symptom burden rather than a diagnostic threshold. These findings support the concept of dolichosigma as part of an anatomic–functional continuum, although causal relationships cannot be inferred. Further studies integrating radiologic and functional assessments are warranted.

**Supplementary Information:**

The online version contains supplementary material available at 10.1007/s00423-026-04015-4.

## Introduction

Constipation is among the most frequent functional gastrointestinal disorders worldwide, affecting approximately 10–14% of the adult population, with a higher prevalence in women and older individuals [1–3]. It is associated with reduced quality of life, impaired work productivity, and health-care utilization comparable to that of chronic systemic diseases such as arterial hypertension or diabetes [4, 5].

Within the spectrum of structural contributors to chronic constipation, dolichosigma, an elongation of the sigmoid colon, has long been recognized, yet its clinical relevance and diagnostic definition remain controversial. In the present study, the term “dolichosigma” refers specifically to elongation of the sigmoid colon, whereas “dolichocolon” is used only when referring to historical or literature-based descriptions of generalized colonic redundancy. Radiologic and cadaveric studies have demonstrated marked interindividual variation in sigmoid length [6, 7], but absolute anatomical reference values derived from direct in vivo measurements in adults have not been established, and the threshold at which elongation becomes clinically significant remains undefined. Mechanistically, an elongated sigmoid loop increases intraluminal transit distance, promotes fecal stasis and mucosal contact time, and facilitates excessive water reabsorption, all of which contribute to hard stool formation and slow-transit constipation [8].

The historical understanding of colonic elongation spans more than two centuries. Early anatomical and radiologic observations described considerable variability in colonic length and suggested that excessive elongation might influence bowel motility [9–15]. Later experimental work confirmed that colonic stretch slows peristalsis, as shown in murine models where distension of the longitudinal muscle layer led to delayed transit [16].

The term “dolichocolon” was introduced by Lardennois and Auborg (1914), who defined it as a colon with a sigmoid loop extending above the line between the iliac crests, a transverse colon descending below this line, and redundant loops at the flexures [17]. This definition remains influential in current anatomical descriptions.

Modern imaging studies have renewed interest in this variant. MRI-based pediatric research demonstrated that children and adolescents with functional constipation exhibit significantly longer colonic or rectosigmoid segments than healthy controls [18], suggesting that elongation may represent a developmental continuum rather than an acquired degenerative change. However, these studies relied on imaging-based estimations rather than direct intraoperative measurements. In adults, dolichosigma frequently coexists with obstructed defecation syndrome or pelvic floor dysfunction, complicating the interpretation of causality [19, 20]. Furthermore, surgical data from women with rectosigmoid endometriosis indicate that dolichocolon may blunt symptomatic relief even after technically successful procedures [21].

Despite this long-standing interest, prospective intraoperative data in unselected adult populations remain scarce. In everyday visceral surgery, dolichosigma is often encountered incidentally, leaving surgeons uncertain whether it represents a benign anatomical variant or a clinically meaningful correlate of functional bowel symptoms.

In contrast to previous cadaveric or radiologic investigations, the present study provides prospective intraoperative in vivo measurements of sigmoid length obtained under standardized laparoscopic conditions in living adult patients. To our knowledge, absolute anatomical reference values derived from direct surgical measurements in adults have not previously been established.

The present prospective two-center study therefore aimed to (i) quantify sigmoid length laparoscopically in adult patients undergoing elective minimally invasive abdominal surgery, (ii) examine its correlation with constipation severity and abdominal pain, and (iii) explore a pragmatic anatomical reference associated with sigmoid elongation in adults.

## Materials and methods

This prospective observational study was conducted between January 2023 and December 2025 and included consecutive adult patients (≥ 18 years) undergoing minimally invasive abdominal surgery at two tertiary surgical centers: St. Elisabeth Hospital Iserlohn (Academic Teaching Hospital of Ruhr-University Bochum) and Marien Hospital Herne (University Hospital of Ruhr-University Bochum). Eligible procedures comprised diagnostic laparoscopy for unclear or acute abdomen, laparoscopic appendectomy, cholecystectomy, minimally invasive inguinal hernia repair, and laparoscopic sigmoid resection for diverticular disease. All patients provided written informed consent for intraoperative measurement of the sigmoid colon and study participation. Ethical approval was obtained from the local ethics committee (registration number 22-7495-BR). This study was conducted and reported in accordance with the STROBE (Strengthening the Reporting of Observational Studies in Epidemiology) guidelines.

Patients were eligible for inclusion if they were at least 18 years of age, underwent a planned laparoscopic or robotic abdominal procedure that allowed adequate visualization of the sigmoid colon, and were able to complete the constipation questionnaires. Patients undergoing open surgery were excluded, as were those with fecal peritonitis, previous colorectal resection involving the sigmoid colon or rectum, obstructing colorectal cancer, or extensive intraabdominal adhesions in which intraoperative measurement would have significantly prolonged operative time or increased surgical risk. The decision to refrain from measurement in such cases was left to the discretion of the operating surgeon. This approach was chosen to ensure patient safety and avoid prolongation of operative time in technically challenging situations.

Laparoscopic measurement technique: after pneumoperitoneum and placement of trocars, the sigmoid colon was identified and gently straightened without traction using predefined anatomical landmarks and without applying longitudinal force. Length was measured from the suspensory attachment at the level of the anterior superior iliac spine (left) to the ventral peritoneal reflection of the sigmoid, using a laparoscopic grasping forceps and a measuring tape that is scaled at 20 cm and can be folded down to 10–5 cm. The same standardized measurement technique was applied at both centers, and all measurements were performed by experienced abdominal surgeons in order to minimize potential interobserver variability.

Care was taken to avoid traction or overstretching of the bowel. Measurements were performed after gentle alignment of the sigmoid loop under physiological pneumoperitoneum conditions to minimize artificial elongation and ensure reproducibility across centers.

Clinical data were recorded prospectively: age, sex, BMI, arterial hypertension (0/1), diabetes mellitus (0 = none, 1 = type 1, 2 = type 2), nicotine abuse (0/1), morphine intake (0/1), psychotropic medication (0/1), self-reported constipation (0/1), abdominal pain during the last 6 months (0/1). In addition, the Wexner Constipation Score (WCS) was obtained in all patients.

For comparative analyses, patients were classified as constipated if constipation was self-reported and/or if the Wexner Constipation Score (WCS) exceeded 5 points. The threshold of WCS > 5 was chosen to reflect clinically relevant symptom burden rather than to serve as a formal diagnostic cutoff. Based on this definition, a constipation group (*n* = 67) and a non-constipation group (*n* = 111) were formed.

Constipation assessment was therefore symptom-based and did not follow formal Rome IV diagnostic criteria, as the primary objective was to evaluate symptom burden in a surgical cohort rather than to establish a gastroenterological diagnosis of functional constipation.

An a priori sample size calculation was performed for the primary analysis assessing the association between sigmoid length and constipation severity as measured by the Wexner Constipation Score. Assuming a small-to-moderate correlation coefficient of *r* = 0.25, a two-sided significance level of 0.05, and a statistical power of 80%, a minimum sample size of 124 patients was required based on Fisher’s z-transformation. To account for potential missing questionnaire data, a 10% increase was applied, resulting in a target sample size of 138 patients. The final study cohort comprised 178 patients and therefore exceeded the calculated requirement.

Continuous variables are presented as mean ± standard deviation and were compared using Welch’s t-test. Categorical variables are expressed as absolute numbers and percentages and were compared using the χ²-test. Receiver operating characteristic (ROC) curve analysis was performed with constipation (yes/no) as the dependent variable and sigmoid length (cm) as the predictor to determine the optimal discriminative cutoff value using the Youden index. In addition, a multivariable logistic regression analysis was performed to assess whether sigmoid length was independently associated with constipation after adjustment for potential confounders. Covariates included age, sex, and BMI based on clinical relevance and observed group differences. Adjusted odds ratios (OR) with 95% confidence intervals (CI) were calculated. All statistical tests were two-sided, and a p-value < 0.05 was considered statistically significant.

## Results

### Patient characteristics

A total of 178 patients were analyzed. The mean age was 56.0 ± 17.1 years, ranging from 20 to 95 years. The mean BMI was 26.4 ± 5.6 kg/m². Eighty-five patients (47.8%) were male and ninety-three (52.2%) female. Arterial hypertension was present in 55 patients (30.9%), diabetes mellitus in 20 (11.2%), and nicotine abuse in 69 (38.8%). Morphine intake was documented in 8 patients (4.5%), and psychotropic medication in 17 (9.6%).

Abdominal pain within the last six months was reported by 135 patients (75.8%). Constipation, defined as self-reported constipation and/or a Wexner Constipation Score (WCS) > 5 points, was present in 67 patients (37.6%) (Table 1).

**Table 1:** Baseline demographic and clinical characteristics of the study population (*n* = 178). Values are presented as mean ± standard deviation or number (%)

Variable	Mean ± SD / *n* (%)
Age (years)	56.0 ± 17.1
BMI (kg/m²)	26.4 ± 5.6
Sex (male)	85 (47.8%)
Arterial hypertension	55 (30.9%)
Diabetes mellitus	20 (11.2%)
Nicotine abuse	69 (38.8%)
Morphine intake	8 (4.5%)
Psychotropic medication	17 (9.6%)
Abdominal pain (6 months)	135 (75.8%)
Constipation (self-report and/or WCS > 5)	67 (37.6%)

### Comparison according to constipation status

When stratified by constipation, constipated patients had a significantly lower BMI compared with non-constipated individuals (25.1 ± 5.4 kg/m² vs. 27.2 ± 5.5 kg/m²; *p* = 0.015). Female sex was markedly overrepresented in the constipation group (73.1% vs. 39.6%; *p* < 0.001).

Arterial hypertension occurred more frequently among constipated patients (41.8% vs. 24.3%; *p* = 0.023), while diabetes mellitus (13.4% vs. 9.9%; *p* = 0.47), nicotine abuse (35.8% vs. 40.6%; *p* = 0.53), morphine intake (6.0% vs. 3.6%; *p* = 0.48), and psychotropic medication (11.9% vs. 8.1%; *p* = 0.44) did not differ significantly.

Abdominal pain was markedly more common in the constipation group, reported by 94.0% vs. 64.9% in non-constipated patients (*p* < 0.001) (Table 2).

**Table 2:** Comparison of demographic and clinical variables by constipation status

Variable	Constipation (*n* = 67)	No constipation (*n* = 111)	*p*-value
Age (years)	55.4 ± 16.8	56.4 ± 17.3	0.71
BMI (kg/m²)	25.1 ± 5.4	27.2 ± 5.5	0.015
Female sex, n (%)	73.1%	39.6%	< 0.001
Arterial hypertension (%)	41.8%	24.3%	0.023
Diabetes mellitus (%)	13.4%	9.9%	0.47
Nicotine abuse (%)	35.8%	40.6%	0.53
Morphine intake (%)	6.0%	3.6%	0.48
Psychotropic medication (%)	11.9%	8.1%	0.44
Abdominal pain (%)	94.0%	64.9%	< 0.001

Comparison of demographic and clinical characteristics between patients with and without constipation. Continuous variables are expressed as mean ± SD and compared using Welch’s t-test; categorical variables are expressed as n (%) and compared using the χ² test

### Sigmoid length and WCS

Mean laparoscopic sigmoid length in the entire cohort was 30.5 ± 12.2 cm (range 10–120 cm). Constipated patients exhibited a significantly longer sigmoid colon compared with non-constipated patients (32.6 ± 12.9 cm vs. 29.2 ± 11.6 cm; *p* = 0.006).

The Wexner Constipation Score (WCS) was, as expected, markedly higher among constipated patients (11.5 ± 5.5 vs. 3.5 ± 3.3; *p* < 0.001). A statistically significant but modest positive correlation between sigmoid length and WCS was observed (*r* = 0.26; *p* < 0.001).

**Fig. 1:**
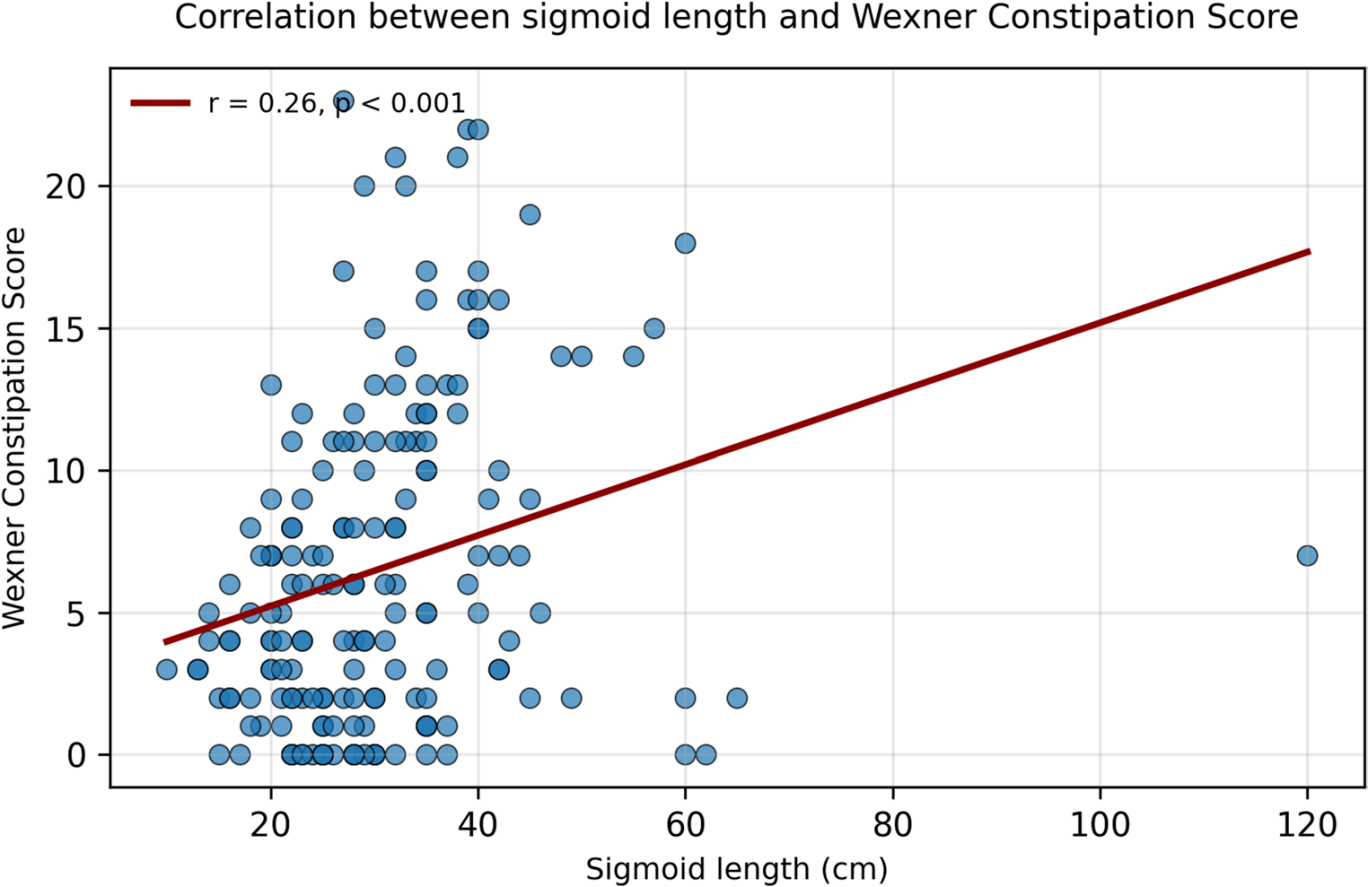
Correlation between sigmoid length and Wexner Constipation Score (WCS). Scatter plot illustrating the positive correlation between laparoscopically measured sigmoid length and Wexner Constipation Score in the study cohort (n = 178). The regression line demonstrates a statistically significant but modest positive association (r = 0.26; p < 0.001), indicating that longer sigmoid segments are related to increased constipation severity

Figure 1 Correlation between sigmoid length and Wexner Constipation Score (WCS).This finding indicates that longer sigmoid segments are associated with more severe constipation symptoms.

### Abdominal pain and sigmoid length

Patients with abdominal pain had significantly longer sigmoid colons compared with those without (31.4 ± 12.5 cm vs. 27.4 ± 10.9 cm; *p* = 0.031).

**Fig. 2:**
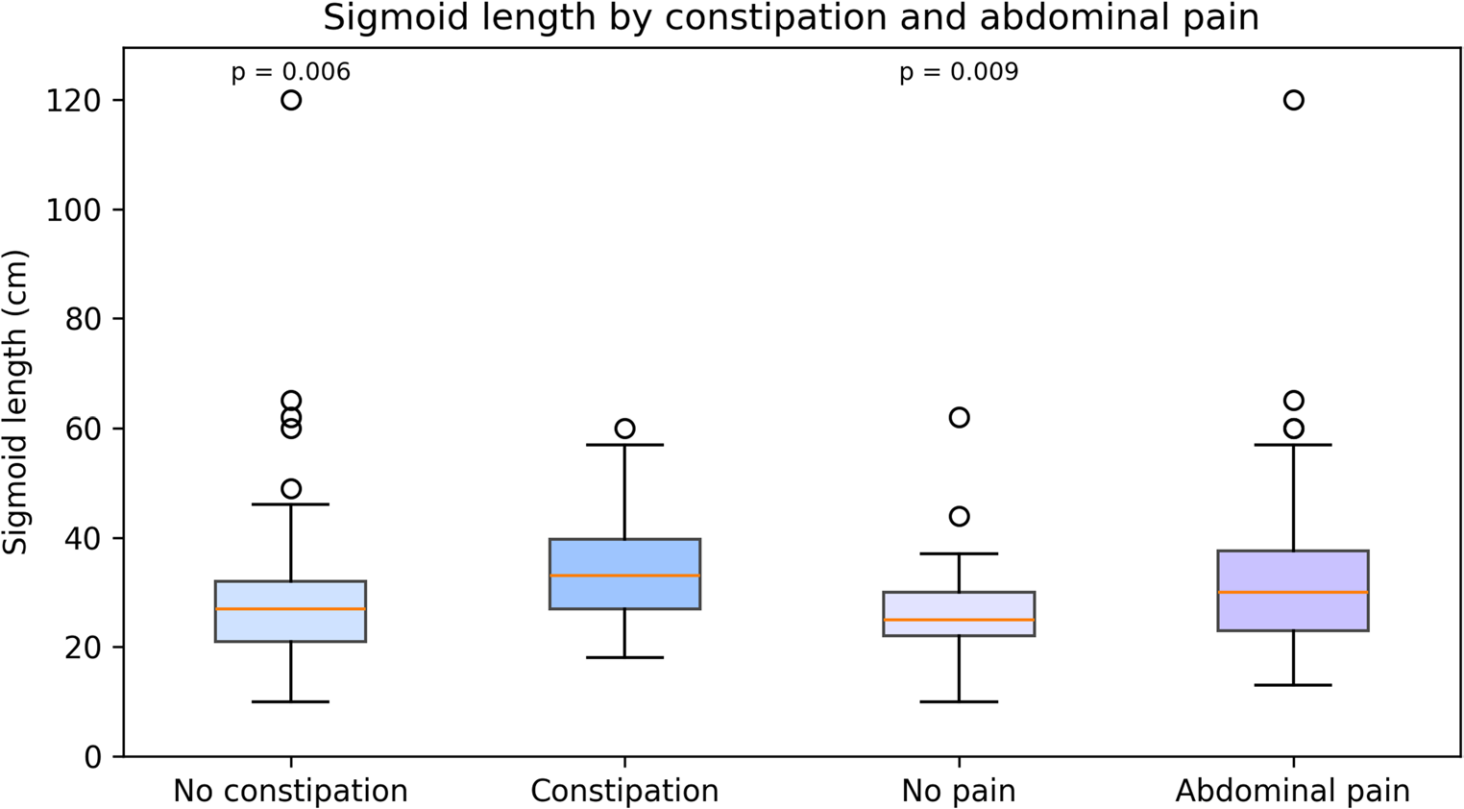
Sigmoid length according to constipation status and presence of abdominal pain. Boxplots comparing laparoscopic sigmoid length between patient subgroups. Constipated patients exhibited significantly longer sigmoid colons than non-constipated patients (32.6 ± 12.9 cm vs. 29.2 ± 11.6 cm; p = 0.006). Similarly, patients with abdominal pain showed longer sigmoids than those without (31.4 ± 12.5 cm vs. 27.4 ± 10.9 cm; p = 0.031). Boxes indicate interquartile ranges with median lines; whiskers represent 1.5× IQR; outliers are shown as dots

These findings suggest that redundancy of the sigmoid colon may be associated not only with constipation but also with abdominal discomfort, supporting the functional relevance of intraoperatively observed sigmoid elongation (Table 3) (Fig. 2).

**Table 3:** Sigmoid length and Wexner Constipation Score (WCS) according to constipation status and presence of abdominal pain. Values are presented as mean ± SD

Parameter		Constipation		No constipation		*p*-value
Sigmoid length (cm)		32.6 ± 12.9		29.2 ± 11.6		0.006
Wexner Constipation Score		11.5 ± 5.5		3.5 ± 3.3		< 0.001
Parameter		Abdominal pain		No abdominal pain		p-value
Sigmoid length (cm)		31.4 ± 12.5		27.4 ± 10.9		0.031

Comparison of sigmoid length and Wexner Constipation Score (WCS) according to constipation and abdominal pain status. Constipated patients had significantly longer sigmoid colons and higher WCS values compared to non-constipated patients. Similarly, patients reporting abdominal pain exhibited longer sigmoid segments than those without pain

### Multivariable logistic regression analysis

To account for potential confounding by demographic and anthropometric variables, a multivariable logistic regression analysis including sigmoid length, sex, BMI, and age was performed. Sigmoid length remained independently associated with constipation (adjusted OR 1.03 per cm increase, 95% CI 1.01–1.06, *p* = 0.019). Female sex was also independently associated with constipation (adjusted OR 4.43, 95% CI 2.21–8.87, *p* < 0.001), whereas BMI showed an inverse association (adjusted OR 0.93, 95% CI 0.87–0.99, *p* = 0.020). Age was not significantly associated with constipation in the adjusted model (Table 4).

**Table 4:** Multivariable logistic regression analysis for factors associated with constipation. Multivariable logistic regression analysis identifying factors independently associated with constipation. OR = odds ratio; CI = confidence interval

Variable	Adjusted OR	95% CI	*p*-value
Sigmoid length (per cm)	1.03	1.01–1.06	0.019
Female sex	4.43	2.21–8.87	< 0.001
BMI (per kg/m²)	0.93	0.87–0.99	0.020
Age (per year)	1.01	0.99–1.03	0.209

*Model statistics:* *n* = 177; Pseudo R² = 0.14; likelihood ratio test *p* < 0.001

### ROC analysis

ROC analysis for discrimination of constipation by sigmoid length yielded an AUC of 0.68. The best cut-off according to the Youden index was 33 cm, providing a sensitivity of 0.52 and a specificity of 0.76. For descriptive clinical interpretation, a range of ≥ 33–35 cm may be considered a pragmatic anatomical reference associated with increased symptom prevalence, as it balances diagnostic performance with anatomical plausibility. However, the moderate AUC indicates limited discriminative ability, and this value should therefore be interpreted as an anatomical reference rather than a diagnostic threshold.

## Discussion

In this prospective laparoscopic study, we evaluated the frequency and clinical implications of sigmoid elongation (dolichosigma) in unselected adult visceral surgery patients. The results demonstrate that sigmoid length varies widely among individuals and that longer sigmoid segments are significantly associated with both constipation severity and the presence of abdominal pain. These findings support the long-standing clinical observation that colonic redundancy, particularly of the sigmoid loop, may represent a relevant anatomical correlate of functional bowel symptoms rather than a purely benign anatomical variant.

Historically, dolichosigma has been interpreted as a congenital or developmental variation of the distal colon. Early anatomic studies already emphasized that the sigmoid colon exhibits greater length variability than any other colonic segment [13, 14]. Later radiologic series and cadaveric measurements confirmed this wide range, typically between 15 cm and over 60 cm in adults [6–8]. However, in contrast to earlier descriptive studies, our laparoscopic data provide in vivo measurements under standardized conditions and suggest that a sigmoid length of approximately 33–35 cm may represent a pragmatic anatomical reference associated with increased symptom prevalence rather than a diagnostic threshold. Beyond this range, the prevalence of constipation and abdominal pain was higher in the present cohort. Unlike earlier definitions based primarily on relative loop position, our findings provide a quantitative anatomical reference derived from direct intraoperative measurements, which may be more applicable to contemporary surgical decision-making.

The association between sigmoid elongation and constipation severity, reflected by a positive correlation between sigmoid length and the Wexner Constipation Score (WCS), is consistent with a potential mechanistic relationship between anatomy and motility. A longer sigmoid colon increases the intraluminal transit distance, facilitating fecal stasis, prolonged mucosal contact, and excessive water reabsorption. This pathophysiologic chain is consistent with experimental observations showing that distension of the colonic wall slows peristaltic activity and transit [16]. An alternative interpretation is that sigmoid elongation may not solely represent a predisposing factor but could also develop as a consequence of chronic stool retention and prolonged distension, given the role of the sigmoid colon as a functional fecal reservoir. The cross-sectional design of our study does not allow conclusions regarding causality. In addition, histopathological findings of partial hypoganglionosis or degenerative changes in elongated colons (Ačkasov 2003) suggest that neuromuscular alterations may coexist, reinforcing the notion that dolichosigma may encompass both structural and functional components [22]. Furthermore, in this study constipation was assessed using a symptom-based approach rather than formal Rome IV criteria, which limits differentiation between functional constipation and secondary or symptom-based constipation in this surgical population.

Interestingly, the present data also reveal a significant relationship between sigmoid length and abdominal pain, even among patients without clinically defined constipation. This observation suggests that elongation may not only delay transit but could also increase mechanical tension, torsion susceptibility, or local distension stimuli within the mesocolon, potentially contributing to visceral hypersensitivity. Similar mechanisms have been discussed in chronic idiopathic constipation and irritable bowel syndrome [23–25]. These associations underline that the relevance of dolichosigma extends beyond stool frequency and should be considered in the broader context of functional abdominal pain syndromes.

Recent pediatric MRI studies provide an important developmental perspective. Children and adolescents with functional constipation already display longer colonic or rectosigmoid segments than healthy peers [18], suggesting that colonic elongation may represent a continuum throughout life rather than an acquired condition in adulthood. In this context, the elongation observed intraoperatively in adults may reflect a combination of congenital predisposition and adaptive remodeling secondary to chronic slow-transit states.

Clinically, dolichosigma often coexists with obstructed defecation syndrome (ODS) and pelvic floor dysfunction, creating diagnostic uncertainty as to whether elongation is primary or secondary [19, 20]. Our findings of a statistically significant but modest positive correlation between objectively measured sigmoid length and constipation severity in an unselected surgical population support the association between constipation and dolichosigma. This aligns with the observation by Driouch et al. (2023) [26], who emphasized the interplay between morphologic redundancy and altered defecatory mechanics.

From a surgical perspective, recognizing an elongated sigmoid colon intraoperatively is clinically relevant. Redundant loops can complicate procedures such as hernia repair, appendectomy, or colectomy, and may predispose to postoperative volvulus or segmental ischemia if left unrecognized. Conversely, sigmoid resection for symptomatic dolichosigma—especially in patients refractory to conservative therapy—has shown favorable outcomes when the entire redundant segment is removed [21, 27]. Our findings may help delineate a pragmatic anatomical reference that can inform such decisions.

Importantly, the association between sigmoid length and constipation persisted after adjustment for potential confounders. In a multivariable logistic regression model including sex, BMI, and age, sigmoid length remained independently associated with constipation. This finding strengthens the hypothesis that sigmoid elongation may represent an anatomical factor associated with bowel dysfunction rather than merely reflecting demographic differences between patient groups. The modest correlation and moderate AUC indicate that sigmoid length alone cannot fully explain constipation pathophysiology, which is multifactorial in nature.

Female sex also showed a strong independent association with constipation, consistent with epidemiological data indicating higher prevalence among women. The inverse association observed with BMI has been described previously and may reflect differences in diet, physical activity, or visceral fat distribution, although causal mechanisms remain uncertain. Overall, these results support the concept that dolichosigma may act as an independent morphological contributor within the multifactorial pathophysiology of constipation.

### Strengths and limitations

The strengths of this study include its prospective two-center design, standardized intraoperative measurements, and the inclusion of unselected patients, providing a realistic picture of sigmoid variability in clinical practice. However, formal assessment of interobserver variability was not performed and therefore represents a limitation. Limitations include the absence of postoperative functional follow-up and the reliance on self-reported symptoms, which may introduce bias. Although multivariable adjustment was performed, residual confounding cannot be excluded due to the observational design. Nonetheless, the observed associations between sigmoid length, constipation, and abdominal pain remain robust and biologically plausible. The absence of a universally validated Wexner cutoff and the combined definition of constipation may influence group classification. Another limitation is the absence of correlation between intraoperative measurements and preoperative imaging modalities such as CT, MRI, or contrast studies. Consequently, the proposed anatomical reference cannot yet be directly translated into non-operative diagnostic settings.

## Conclusion

In conclusion, sigmoid elongation beyond approximately 33–35 cm appears to represent a morphologic marker associated with increased prevalence of constipation and abdominal pain rather than an incidental anatomical variant. The findings support a concept of dolichosigma as part of an anatomic–functional continuum, although causal relationships cannot be inferred from this cross-sectional study. A sigmoid length of approximately ≥ 33–35 cm may therefore serve as a pragmatic anatomical reference associated with symptom burden rather than a diagnostic threshold. Further multicenter studies integrating radiologic, functional, and neurohistological assessments are warranted to standardize definitions and clarify the developmental versus acquired nature of this entity.

## Supplementary Information

Supplementary Material 1.

Supplementary Material 2.

## Data Availability

The data that support the findings of this study are available from the corresponding author upon reasonable request.

## References

[1] Black CJ, Ford AC (2018) Chronic idiopathic constipation in adults: epidemiology, pathophysiology, diagnosis and clinical management. Med J Aust 209:86–91. 10.5694/mja18.00241 29996755 10.5694/mja18.00241

[2] Suares NC, Ford AC (2011) Prevalence of, and risk factors for, chronic idiopathic constipation in the community: systematic review and meta-analysis. Am J Gastroenterol 106:1582–1591 quiz 1581, 1592. 10.1038/ajg.2011.164 21606976 10.1038/ajg.2011.164

[3] Wald A, Scarpignato C, Mueller-Lissner S et al (2008) A multinational survey of prevalence and patterns of laxative use among adults with self-defined constipation. Aliment Pharmacol Ther 28:917–930. 10.1111/j.1365-2036.2008.03806.x 18644012 10.1111/j.1365-2036.2008.03806.x

[4] Wald A, Scarpignato C, Kamm MA et al (2007) The burden of constipation on quality of life: results of a multinational survey. Aliment Pharmacol Ther 26:227–236. 10.1111/j.1365-2036.2007.03376.x 17593068 10.1111/j.1365-2036.2007.03376.x

[5] Sun SX, Dibonaventura M, Purayidathil FW et al (2011) Impact of chronic constipation on health-related quality of life, work productivity, and healthcare resource use: an analysis of the National Health and Wellness Survey. Dig Dis Sci 56:2688–2695. 10.1007/s10620-011-1639-5 21380761 10.1007/s10620-011-1639-5

[6] Alatise OI, Ojo O, Nwoha P et al (2013) The role of the anatomy of the sigmoid colon in developing sigmoid volvulus: a cross-sectional study. Surg Radiol Anat 35:249–257. 10.1007/s00276-012-1037-5 23143017 10.1007/s00276-012-1037-5

[7] Bhatnagar BNS, Sharma CLN, Gupta SN et al (2004) Study on the anatomical dimensions of the human sigmoid colon. Clin Anat 17:236–243. 10.1002/ca.10204 15042573 10.1002/ca.10204

[8] Raahave D (2018) Dolichocolon revisited: An inborn anatomic variant with redundancies causing constipation and volvulus. World J Gastrointest Surg 10:6–12. 10.4240/wjgs.v10.i2.6 29492185 10.4240/wjgs.v10.i2.6 PMC5827035

[9] Monterossi P (1820) Über widernatürliche Biegungen des Dickdarms als Ursache des Todes neugeborener Kinder. Deutsches Archiv für Physiologie 1820:556–571

[10] Kienböck R (1913) Ueber das Sigma elongatum mobile (Röntgenbefund). Münchener Medizinische Wochenschrift 1913:68–70

[11] Shober JB (1898) Anomalous positions of the colon. Am J Med Sci 1898:405–419. 10.1097/00000441-189810000-00003

[12] Black CE (1912) Displacements of the colon. Ann Surg 1912:888–899. 10.1097/00000658-191212000-00011 10.1097/00000658-191212000-00011 PMC1407439 17862941

[13] Bryant J (1924) Observations upon the growth and length of the human intestine. Am J Med Sci 1924:499–520. 10.1097/00000441-192404000-00003

[14] Treves F (1885) Lectures on the anatomy of the intestinal canal and peritoneum in man. BMJ 1885:415–419 10.1136/bmj.1.1261.415 PMC2255844 20751176

[15] Kantor JL (1931) Common anomalies of the duodenum and colon: Their practical significance. JAMA 1931:1785–1790

[16] Heredia DJ, Dickson EJ, Bayguinov PO et al (2010) Colonic elongation inhibits pellet propulsion and migrating motor complexes in the murine large bowel. J Physiol 588:2919–2934. 10.1113/jphysiol.2010.191445 20547675 10.1113/jphysiol.2010.191445 PMC2956907

[17] Lardennois G AP (1914) Allongements segmentaires du gros intestin: les dolichocolies. J de Radiol et d’Électrologie 1914:65–74

[18] Sharif H, Hoad CL, Abrehart N et al (2024) Colon length in pediatric health and constipation measured using magnetic resonance imaging and three dimensional skeletonization. PLoS ONE 19:e0296311. 10.1371/journal.pone.0296311 38165858 10.1371/journal.pone.0296311 PMC10760671

[19] Driouch J, Sajid S, Bausch D et al (2025) Robot-assisted mesh rectosacropexy and sigmoid colon resection for obstructive defecation syndrome: a two-stage surgical approach. J Robot Surg 19:123. 10.1007/s11701-025-02283-8 40119229 10.1007/s11701-025-02283-8

[20] Rao SSC, Patcharatrakul T (2016) Diagnosis and Treatment of Dyssynergic Defecation. J Neurogastroenterol Motil 22:423–435. 10.5056/jnm16060 27270989 10.5056/jnm16060 PMC4930297

[21] Raimondo D, Mattioli G, Casadio P et al (2020) Frequency and clinical impact of Dolichocolon in women submitted to surgery for rectosigmoid endometriosis. J Gynecol Obstet Hum Reprod 49:101697. 10.1016/j.jogoh.2020.101697 32018043 10.1016/j.jogoh.2020.101697

[22] Ačkasov SI (2003) Anomalies of development and position of the colon: clinic, diagnosis, treatment. Moscow. Russian State Medical University, Russia

[23] Southwell BR (2010) Colon lengthening slows transit: is this the mechanism underlying redundant colon or slow transit constipation? J Physiol 588:3343. 10.1113/jphysiol.2010.196121 20843833 10.1113/jphysiol.2010.196121 PMC2988498

[24] Picciariello A, Rinaldi M, Grossi U et al (2022) Time trend in the surgical management of obstructed defecation syndrome: a multicenter experience on behalf of the Italian Society of Colorectal Surgery (SICCR). Tech Coloproctol 26:963–971. 10.1007/s10151-022-02705-x 36104607 10.1007/s10151-022-02705-x PMC9637616

[25] Prandota J, Iwańczak F, Pytrus T (2003) Zmiany połozenia i długości poprzecznicy powodujace bóle brzucha i przewlekłe zaparcia w wieku dojrzewania (Changes of the position and length of the transverse colon causing abdominal pain and chronic constipation during adolescence). Pol Merkur Lekarski 15:47–50 14593959

[26] Driouch J, Thaher O, Brinkmann S et al (2023) Robotic-assisted rectosigmoid resection rectopexy with natural orifice specimen extraction (NOSE): technical notes, short-term results, and functional outcome. Langenbecks Arch Surg 408:177. 10.1007/s00423-023-02918-0 37140719 10.1007/s00423-023-02918-0

[27] Rudroff C, Madukkakuzhy J, Hernandez AV et al (2024) Early safety and efficiency outcomes of a novel interdisciplinary laparoscopic resection rectopexy combined with sacrocolpopexy for women with obstructive defecation syndrome and pelvic organ prolapse: a single center study. BMC Surg 24:185. 10.1186/s12893-024-02474-4 38877450 10.1186/s12893-024-02474-4 PMC11177501

