# Supplementary material for: Prospective laparoscopic assessment of sigmoid elongation: association with constipation and abdominal pain
Source: Langenbecks Arch Surg. 2026 Mar 25;411(1):114. doi: 10.1007/s00423-026-04015-4 (PMC13053446; doi:10.1007/s00423-026-04015-4)
Supplement: Supplementary file 1 — Supplementary Material 1. [file 423_2026_4015_MOESM1_ESM.docx]

**STROBE Statement—Checklist of items that should be included in reports of observational studies**

| **Item No.** | **Recommendation** | **Reported on Page** |
| --- | --- | --- |
| **Title and Abstract** |  |  |
| 1 | Indicate the study design with a commonly used term in the title or abstract | Title, Abstract |
|  | Provide an informative and balanced summary of what was done and what was found | Abstract |
| **Introduction** |  |  |
| 2 | Explain the scientific background and rationale for the investigation | Introduction |
| 3 | State specific objectives and hypotheses | End of Introduction |
| **Methods** |  |  |
| 4 | Present key elements of study design early in the paper | Methods |
| 5 | Describe the setting, locations, and relevant dates | Methods |
| 6 | Give eligibility criteria and sources/methods of participant selection | Methods |
| 7 | Clearly define outcomes, exposures, predictors, confounders | Methods |
| 8 | For each variable, give data sources and measurement details | Methods |
| 9 | Describe efforts to address potential sources of bias | Methods, Discussion |
| 10 | Explain how study size was determined | Methods (sample size calculation) |
| 11 | Explain handling of quantitative variables | Statistical analysis |
| 12 | Statistical methods, including confounder control | Statistical analysis, Results |
| **Results** |  |  |
| 13 | Report numbers of individuals at each stage | Results |
| 14 | Descriptive data of participants | Table 1 |
| 15 | Outcome data | Results, Tables |
| 16 | Main results with estimates and precision | Results, Tables |
| 17 | Other analyses (e.g., ROC, multivariable analysis) | Results |
| **Discussion** |  |  |
| 18 | Summarize key results with reference to objectives | Discussion |
| 19 | Discuss limitations, bias, imprecision | Discussion |
| 20 | Provide cautious overall interpretation | Discussion |
| 21 | Discuss generalizability | Discussion |
| **Other Information** |  |  |
| 22 | Funding and ethical approval | Methods / Ethics statement |
